# Influence of the soil sealing on the geoaccumulation index of heavy metals and various pollution factors

**DOI:** 10.1007/s11356-016-8209-5

**Published:** 2016-12-16

**Authors:** Przemysław Charzyński, Andrzej Plak, Agnieszka Hanaka

**Affiliations:** 1 Faculty of Earth Sciences, Department of Soil Science and Landscape Management, Nicolaus Copernicus University in Toruń, Lwowska St. 1, 87-100 Toruń, Poland; 2 Faculty of Earth Sciences and Spatial Management, Department of Soil Science and Protection, Maria Curie-Skłodowska University, Kraśnicka Ave. 2cd, 20-718 Lublin, Poland; 3 Faculty of Biology and Biotechnology, Department of Plant Physiology, Maria Curie-Skłodowska University, Akademicka St. 19, 20-033 Lublin, Poland

**Keywords:** Ekranic Technosols, Enrichment factor, Geochemical load index, Heavy metals, Pollution assessment, Pollution load index, Soil sealing, Urban soils

## Abstract

Soil sealing belongs to the most destructive and damaging processes to the soil environment. Soil sealing interrupts or greatly restricts the exchange of matter and energy between the biosphere, hydrosphere, and atmosphere and the soil environment. The aim of this study was to compare the content of heavy metals (Cd, Cr, Cu, Hg, Fe, Ni, Pb, Zn) of Ekranic Technosols by applying indicators such as geoaccumulation index (*I*_geo_), enrichment factor (EF), and pollution load index (PLI), which allowed to determine quantitatively the impact of the soil sealing degree on the content of heavy metals and to distinguish natural from anthropogenic sources of origin of heavy metals. In general, 42 soils from different parts of the city of Toruń (NW Poland) were sampled and divided into three groups according to the degree of soil sealing: completely sealed with asphalt or concrete (A), semi-permeable (partially sealed with cobblestones and concrete paving slabs (B)), and reference (non-sealed) (C). The results indicate that the artificial sealing in urban areas slightly affects the content of heavy metals in soils. However, based on PLI, *I*_geo_, and EF, it was found that the sealing has influence on soil properties and unsealed soil is the most exposed to the accumulation of pollutants.

## Introduction

Understanding the complexity of the functioning of soil systems and the interaction with human activity is particularly important in urban areas. Due to a continuous human existence and activities, these soils are very often truncated or buried under transported material, as well as highly compacted and contaminated. As a consequence, their hydrological characteristics, degree of compaction and disturbances, and relocations of material from original horizons are extremely different from those of natural soil types.

The concentration of heavy metals in soils, associated with litogenesis and pedogenesis, depends on the mineralogical composition of parent material and the direction and pace of the process of soil formation, which determines the distribution of trace elements in the soil profile (Luo et al. 2012a; Wong et al. 2006). Moreover, technogenic activity applies all of the abovementioned processes. The study of heavy metal deposition and accumulation is of increasing interest because of the awareness that heavy metals present in soil may have negative consequences on the human health and whole environment. Heavy metals may enter into aquatic ecosystems from anthropogenic sources, such as industrial waste water discharges, sewage waste water, fossil fuel combustion, and atmospheric deposition. Urban soils are changed as a result of activity of various processes, i.e., sealing, compaction, storage, and mixing (Plak et al. 2015; Wei et al. 2013). Sealing the soil belongs to the most destructive processes to the soil environment. In principle, it is an irreversible process, which is defined as the destruction of soil cover by partial or total application of the soil impermeable layer. Total sealing of the soil in urban areas is caused by concrete or asphalt (Siebielec et al. 2015). Soils can also be sealed with a semi-permeable surface, e.g., concrete paving, which allows partial penetration of air and water (Nestroy 2006) and allows saving the certain features of soil (Piotrowska-Długosz and Charzyński 2015). In the European Union, on average 51% of artificial surfaces are sealed, but the composition varies considerably among Member States, depending on the dominant settlement structures and the intensity of the interpretation of artificial surfaces (Siebielec et al. 2015). In urban areas, as a direct result of human activity, the impact of industrial and municipal building, industry and communication, and artifacts deposited in soil, heavy metals are present in high concentrations (Charzyński et al. 2013; Madrid et al. 2004; Puskas and Farsang 2009; Xia et al. 2011).

In order to determine the degree of contamination of surface soil levels with heavy metals, their concentration is compared with the content of the local geochemical background, or by calculation of geoaccumulation index (*I*_geo_), enrichment factor (EF), or pollution load index (PLI) (Horckmans et al. 2005; Muller 1969; Tomlinson et al. 1980). The main factors influencing the distribution of heavy metals in the soil profile are the content of humus and iron and manganese oxides, as well as texture, soil pH, and the rinsing processes, accompanied by the movement of water within the soil profile (Luo et al. 2012b; Madrid et al. 2008). Metals of anthropogenic origin are more mobile than those of pedogenic origin. In urban areas, the nature of the metals accumulation and their genesis in the soil is often ambiguous, and all the abovementioned processes, which are widely described in the literature, are distorted when the soil is sealed (Kabata-Pendias and Pendias 1999; Reimann et al. 2005; Sauerwein 2011; Siebielec et al. 2015).

The aim of this study was to compare the content of heavy metals (Cd, Cr, Cu, Hg, Ni, Pb, Zn) of Ekranic Technosols from Toruń, Poland, and adjacent, non-sealed soils. We assumed that the soils sealed for 30 to 40 years affect the diversity of heavy metal content in two separate groups of the completely and partially sealed soils while compared with the non-sealed soil. Moreover, we assumed that the use of indicators such as *I*_geo_, EF, and PLI allows to determine quantitatively the impact of the soil sealing on the content of heavy metals.

The problem of uncontrolled propagation of the sealed surface in Poland, especially in the cities, is remotely explored, and the processes occurring in the sealed soils are not comprehensively studied. Moreover, few researchers in the world do their research on geochemical environment of the sealed soil, focusing primarily on the changes in the physical properties or biological activity (lithium) (Greinert 2015; Puskás and Farsang 2009; Zhao et al. 2012). The presented studies show the unique issues related to the impact of (completely or partially) sealed soil on disturbance of heavy metal circulation, which is of great importance in terms of quality of life and health of urban residents, the condition of the environment in cities, and the penetration of pollutants into groundwater. The research material gives extensive knowledge about the impact of the sealing process on soil geochemistry.

## Materials and methods

### Description of the study area and soil sampling

**Fig. 1:**
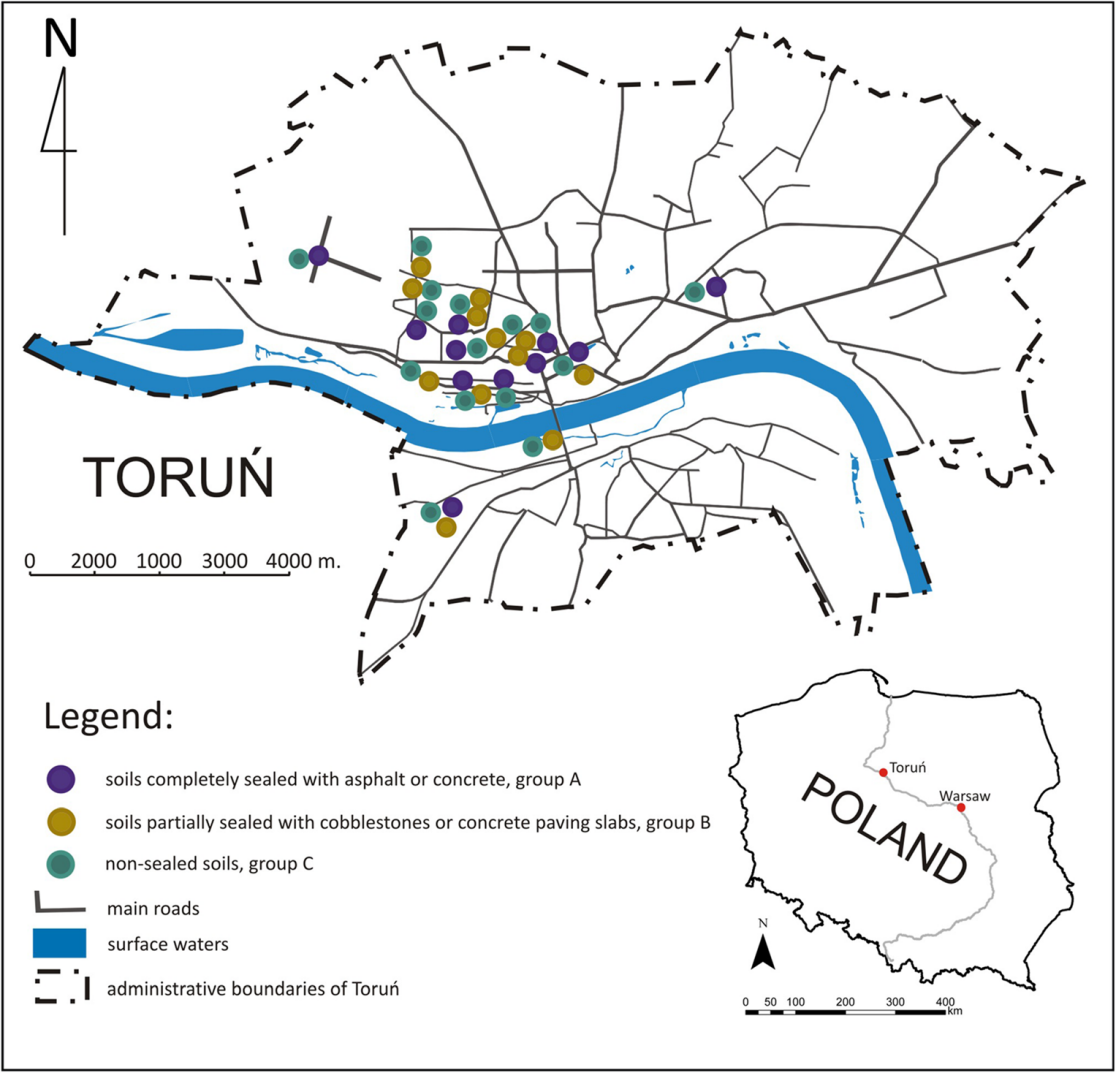
Location of study sites in Toruń, Poland

The study sites were located within the area of Toruń (18.609° E, 53.020° N), the city in the North West Poland (Fig. 1). Majority of natural and technogenically transformed soils in the studied area have texture of medium sand according to USDA Soil Taxonomy (Soil Survey Staff 2014), but some have texture of coarse and fine sand. The moderate climate of the region is between the marine type of Western Europe and the continental type of Eastern Europe. The average annual temperature for the whole year was 8.4 °C, while the annual rainfall was 535 mm (Source: data of the Institute of Meteorology and Water Management: stat.gov.pl).

**Fig. 2:**
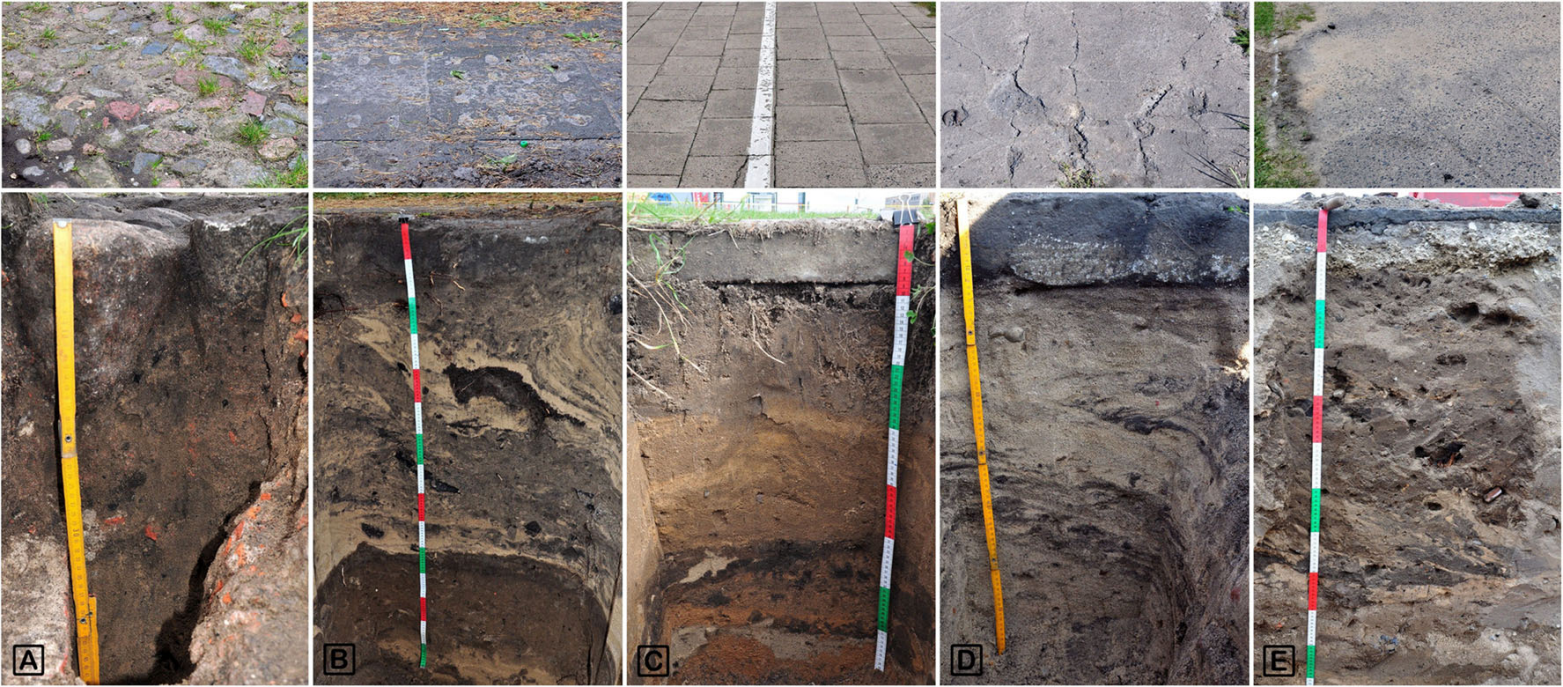
Examples of sealed soils. Partially sealed soils: A—profile no. 41; B—profile no. 6; C—profile no. 28; completely sealed soils: D—profile no. 19; E—profile no. 5

In general, 42 soils from various parts of the city (Table 1) were sampled between April of 2012 and September 2013 (Fig. 1). In each place, soils sealed with asphalt or concrete, fulfilling criteria of technic hard material (IUSS Working Group WRB 2015) (completely sealed, group A; 15 samples), semi-permeable materials (cobblestones and concrete paving slabs, partially sealed, group B; 11 samples), and reference (non-sealed, group C; 16 samples) soils were selected (Fig. 2). They were situated in direct proximity to ensure similar history of technogenic disturbances, the same environmental conditions, and soil texture. Samples from both categories of sealed soils were collected from the topmost original horizon that was left after the process of construction of sealing surface. Soil samples were collected from a depth of 15–25 cm or 10–20 cm. Depth of sampling depended on the thickness of the technic hard material combined with the ballast layer used for stabilization of the pavement construction. Reference sites without sealing were located on lawns of green-belts next to pavements or roads (about 1 m) to ensure maximum soil comparability. Samples were collected from the same depth as in the sealed soils. The sealing was performed between 30 and 40 years ago, in 1970 or 1980.

**Table 1:** Sampling sites and soil profiles description

Profile no.	Sampling site description	Soil profile description
1	Bydgoski park; close to the corner of Konopnicka and Rybaki streets	City park, walkway; sealed with asphalt
2		Sidewalk; sealed with concrete setts, 30 × 30 cm
3		City park, grass lawn; control for profile nos. 1 and 2
4	Zajęcze Góry park (sand dunes covered mainly by Scots Pines); close to the corner of Bema and Gałczyński streets	Walkway; sealed with asphalt
5		Walkway; sealed with asphalt (on the other side)
6		Access street; sealed with concrete slabs 1 × 1 m with holes 5 × 10 cm
7		Sidewalk; sealed with small concrete setts, so-called polbruk
8		Roadside greenbelt, grass; control for profile nos. 4, 5, 6, and 7
9	Bielany suburb; next to Krzemieniecka street	Roadside; sealed with solid concrete slabs, 30 × 30 cm
10		Grass plot; control for profile no. 8
11	Chełmińskie suburb; recreation area (playground, basketball field) next to Sportowa street	Walkway; sealed with asphalt
12		Sidewalk; sealed with concrete setts, 30 × 30 cm
13		Grass plot; control for profile no. 8
14	Nicolaus Copernicus University in Toruń Campus; recreational area next to dormitories buildings	Walkway; sealed with concrete with thin layer of asphalt on the top
15		Walkaway; sealed with concrete setts, 40 × 40 cm
16		Walkaway; sealed with small concrete setts, so-called polbruk
17		Grass plot, some pines; control for profile nos. 10 and 11
18	Jordanki area; former recreational area, car park	Square covered with asphalt
19		Square covered with large concrete slabs 1 × 2 m
20		Bare ground, control for profile nos. 18 and 19
21	Chełmińskie suburb; near Szosa Chełmińska street	Sidewalk, granite slabs 70 × 70 cm
22		Grass plot; control for profile no. 21
23	Vistula river left bank flood plain	Access street to Dybowski castle; sealed with cobblestones
24		Meadow with bushes; control for profile nos. 13 and 14
25	Bydgoskie suburb; next to Bydgoska street	Walkway; sealed with concrete
26		Roadside greenbelt, grass; control for profile no. 25
27	Na Skarpie suburb; constructed in ‘70 of XX cent., next to Lubicka street (major, busiest city thoroughfare), eastern part of city	Walkway; sealed with asphalt
28		Walkaway; sealed with solid concrete slabs, 30 × 30 cm
29		Roadside greenbelt, grass; control for profiles nos. 27 and 28
30	Rubinkowo suburb; constructed in ‘70 of XX cent.; next to Łyskowskiego street, area between four-storey apartment buildings, eastern part of the city	Walkway; sealed with asphalt
31		Grass plot with some trees; control for profile no. 30
32	Toruń airport area; operated by Pomeranian Flying Club, western part of the city	Runaway; sealed with concrete slabs, 3 × 3 m
33		Meadow; control for profile no. 16
34	Podgórz suburb; left bank part of the city	Access street; sealed with solid concrete slabs
35		Access street; sealed with concrete slabs 1 × 1 m with holes 5 × 10 cm,
36		Grass plot, control for profile nos. 18 and 19
37	Bielany suburb, next to Okrężna street	Roadside; sealed with solid concrete slabs, 30 × 30 cm
38		Roadside greenbelt; control for profile no. 21
39	Bielany suburb; next to Krzemieniecka street	Roadside; sealed with concrete slabs,1 × 0.75 m with holes 5 × 10 cm
40		Roadside greenbelt, grass; control for profile no. 24
41	Yard of end of XIX cent. house; the corner of Konopnicka and Rybaki streets	Yard of nineteenth century house; sealed with cobblestone
42		Grass lawn; control for profile nos. 1 and 2

### Chemical and physical analysis of the soil

After air-drying at room temperature and sieving (<2 mm), the soil samples were analyzed for physical and chemical properties. Physicochemical properties were determined using standard methods (Van Reeuwijk 2002). Each soil sample was analyzed in triplicate. Texture was assessed by the areometric method combined with the sieve method. The pH in 1 mol l^−1^ KCl was measured using the potentiometric method in 1:2.5 soil:solution suspensions; total organic carbon (OC_TOT_) and total nitrogen (N_TOT_) content were determined using a dry combustion CN analyzer (Vario Max CN). Available phosphorus (P) was assayed using the vanadium-molybdenum method.

### Heavy metal analysis

The total content of heavy metals (denoted by _TOT_) in the soil samples was determined using the ICP MS technique before dissolving with aqua regia.

### Data analysis

*I*_geo_ (Muller 1969) is computed using the following equation:

$$ {I}_{geo}={ \log}_2\left[{C}_n/1.5\times {B}_n\right], $$

where *C*_n_ is the measured concentration of the element in environment and *B*_n_ is the geochemical background value in soil.

According to Muller (1969), the *I*_*g*eo_ for each metal is calculated and classified as uncontaminated (*I*_geo_ ≤ 0); uncontaminated to moderately contaminated (0 < *I*_geo_ ≤ 1); moderately contaminated (1 < *I*_geo_ ≤ 2); moderately to heavily contaminated (2 < *I*_geo_ ≤ 3); heavily contaminated (3 < *I*_geo_ ≤ 4); heavily to extremely contaminated (4 < *I*_geo_ ≤ 5); and extremely contaminated (*I*_geo_ ≥ 5).

The EF calculation is expressed below as

$$ EF={\left[{C}_{\mathrm{x}}/{C}_{ref}\right]}_{\mathrm{sample}}/{\left[{C}_{\mathrm{x}}/{C}_{ref}\right]}_{\mathrm{background}}, $$

where *C*_x_ is the concentration of the element of interest and *C*_ref_ is the concentration of reference element for normalization.

Enrichment factors of heavy metals were calculated for each soil sample relative to the background values of abundance of chemical elements in the local parent rock, choosing Fe as the reference element (according to Kabata-Pendias and Pendias (1999) −12.9 g kg^−1^ Fe).

Geochemical background was determined on the basis of Czarnowska (1996), Kabata-Pendias and Pendias (1999), Lis and Pasieczna (1995) and Pasieczna (2003). There have been analyzed uncultivated areas, taking into account the different geological provinces according to classification proposals used for geochemical map of the world (Damley 1995). The area of Toruń is situated in the northern province of the Polish Lowland, where sand is mostly present.

Five contamination categories are recognized on the basis of the enrichment factor: EF < 2 states deficiency to minimal enrichment, EF = 2–5—moderate enrichment, EF = 5–20—significant enrichment, EF = 20–40—very high enrichment, and EF > 40 extremely high enrichment (Duzgoren-Aydin et al. 2006; Sezgin et al. 2003).

PLI (Tomlinson et al. 1980) is expressed as follows:

$$ PLI={\left({CF}_{Cr}\times {CF}_{Ni}\times {CF}_{Cu}\times {CF}_{Zn}\times {CF}_{Cd}\times {CF}_{Pb}\times {CF}_{Hg}\right)}^{1/7}, $$

where CF is the contamination factor obtained by calculating between each metal’s concentration and its background value.

When PLI is greater than 1, it means that contamination exists; however, if PLI is less than 1, there is no metal contamination.

### Statistical data evaluation

Data were analyzed using non-parametric tests (Statistica 6.1, Stat Soft. Inc.). Moreover, principal component analysis (PCA) of two factors and cluster analysis (CA) using the Euclidean distance were applied.

## Results and discussion

### The basic properties of soils

The physical and chemical properties of the studied soils are presented in Table 2. The studied soils represented poorly diversified texture distribution, and according to the USDA, they represented mainly sand and loamy sand. In the studied profiles, there was a significant presence of the skeleton, which was mostly the building rubble.

**Table 2:** Physical and chemical properties of urban soils in Toruń

		Sand	Silt	Clay	pH_KCl_	OC_TOT_	*N*_TOT_	*P*_AV_
		(%)				(g kg^−1^)		(mg kg^−1^)
Gr. A	Mean	97.4 ± 3.4	2.3 ± 2.7	0.4 ± 0.7	7.6 ± 1.15	2.79 ± 1.67	0.17 ± 0.09	180 ± 127
	Range	91–100	0–7	0–2	4.6–8.4	1.79–5.10	0.024–0.32	47–460
Gr. B	Mean	98.5 ± 2.0	1.3 ± 1.4	0.2 ± 0.6	7.5 ± 1.13	2.22 ± 1.81	0.15 ± 0.07	98 ± 31
	Range	93–100	0–5	0–2	5.6–8.6	1.31–4.63	0.064–0.30	57–154
Gr. C	Mean	95.5 ± 4.5	3.8 ± 3.5	0.2 ± 0.6	7.6 ± 0.6	5.60 ± 2.36	0.36 ± 0.17	200 ± 138
	Range	88–99	1–9	0–2	6.7–8.3	2.24–9.90	0.13–0.72	50–456

Gr. A—completely sealed soil, Gr. B—partially sealed soil, Gr. C—non-sealed soil. Values are means ± SD

There is no difference between the soil groups in the average pH values (Table 2). Soil sealing significantly reduced the content of OC_TOT_ and N_TOT_ compared to non-sealed soil. The C:N ratio ranged widely from 9.3 to 28.9 and was not significantly different for the reference and sealed soils, or for both categories of soil sealing (Table 2). Distribution of organic carbon content showed high variability, which is quite typical for the anthropogenic soil (e.g., replenishing a new layer or mixing of soil material) (Greinert 2015). Higher levels of OC_TOT_ and N_TOT_ in non-sealed soils in relation to other comparable groups can be primarily explained by the lack of barriers in the supply of organic matter but also higher biological activity in the non-sealed soil. The content of available phosphorus, *P*_AV_, also showed large range reaching from 47 to 456 mg kg^−1^, but no clear trend appears between the compared groups of soil.

### Relation of soil sealing and heavy metal content

Heavy metal concentrations in urban soils in Toruń are given in Table 3. The total content of heavy metals (Cd, Cr, Cu, Ni, Pb, Zn) in soils of all groups does not exceed the size of the content permitted in Poland (Regulation of the Minister of Environment on soil quality standards and earth quality standards, Dz.U.02.165.1359 dated 04.10.^2002^). The results were also compared with the permissible content of heavy metals in the soil in the Netherlands and Germany, and no exceeded values were detected (NGG 1995, BBodSchV, 1999).

**Table 3:** Heavy metal concentrations in urban soils in Toruń

		Cu	Pb	Zn	Ni	Cd	Cr	Hg
		(mg kg^−1^)						
Gr. A	Mean	11.8 ± 15.3	25.2 ± 40.4	23.7 ± 21.5	3.0 ± 2.0	0.1 ± 0.3	2.7 ± 1.5	0.11 ± 0.2
	Range	1.2–56.5	2.8–159.2	4.5–69.5	0.7–7.4	0.01–1.2	1.2–5.7	0.01–0.6
Gr. B	Mean	3.0 ± 1.7	7.9 ± 6.0	16.5 ± 9.0	2.2 ± 1.2	0.1 ± 0.2	2.4 ± 1.0	0.03 ± 0.03
	Range	1.3–6.6	2.4–21.4	6.3–35.5	0.9–5.0	0.02–0.9	1.1–4.3	0.01–0.1
Gr. C	Mean	9.5 ± 9.5	25.1 ± 29.5	41.5 ± 45.8	3.5 ± 2.0	0.1 ± 0.1	3.6 ± 2.2	0.1 ± 0.2
	Range	1.6–30.9	3.3–100.7	9.5–151.6	0.8–6.5	0.03–0.4	1.1–9.0	0.01–0.7
Background^a^		7.10	9.80	30.00	10.20	0.18	27.00	0.06

Gr. A—completely sealed soil, Gr. B—partially sealed soil, Gr. C—non-sealed soil. Values are means ± SD

^a^(Czarnowska 1996; Kabata-Pendias and Pendias 1999; Lis and Pasieczna 1995; Pasieczna 2003)

Analysis of heavy metal content in the three soil groups studied in Toruń arranged soil in the following order: group C (non-sealed) > group A (completely sealed) > group B (partially sealed) (Table 3).

The level of geochemical background of heavy metals in relation to the completely sealed soil exhibits higher values for Cu, Pb, Zn, and Hg (Table 3). The highest concentration of heavy metals in all examined soil groups was detected in the industrial zone of the city and in the locations along roads with heavy traffic. Content of heavy metals in partly sealed soils showed content comparable to the geochemical background. The average content of Cu, Pb, Zn, Cd, and Hg shows higher values compared with the average value of geochemical background in Poland. Concentration values for the other elements, i.e., Ni and Cr, were comparable.

Comparison of heavy metal content in sealed soils with other cities in Poland, Germany, Hungary, and Russia shows that the concentration of the analyzed elements in the urban soils of Toruń is on a medium level (Table 4).

**Table 4:** Mean heavy metal concentrations in topsoil of sealed urban soils from some European cities

	Cr_TOT_	Zn_TOT_	Cd_TOT_	Pb_TOT_	Cu_TOT_	Hg_TOT_	Ni_TOT_	Reference
	(mg kg^−1^)							
Toruń, Poland	2.73	23.75	0.15	25.23	11.8	0.12	3.05	This study
Szczecin, Poland	n.a.	42.6	0.28	29.1	25.8	n.a.	21.8	Sammel et al. 2013
Szczecin, Poland	n.a.	28.8	0.29	18	8.07	n.a.	8.17	Meller et al. 2013
Zielona Góra, Poland	n.a.	293	0.58	85.2	33	n.a.	9.7	Greinert 2013
Debrecen, Hungary	8	67.7	<1	10.3	7.1	n.a.	4.58	Sandor et al. 2013
Hannover, Germany	n.a.	186	1.2	172	52	n.a.	20	Wesolek 2008
Moscow, Russia	n.a.	58.2	0.3	74.2	24.1	n.a.	1.9	Stroganova et al. 1998
Rostov, Russia	84.2	68.8	n.a.	15.9	28.0	n.a.	25.2	Bezuglova et al. 2016

_*TOT*_ total, *n.a.* data not available

The *I*_geo_ index is used to assess heavy metal contamination in urban soils by comparing current and pre-industrial concentrations, although it is not always easy to reach pre-industrial sediment layers. It is also employed in pollution assessment of heavy metals in urban road dust.

In this study, the background geochemical composition of the city soil types (Czarnowska 1996; Kabata-Pendias and Pendias 1999; Lis and Pasieczna 1995; Pasieczna 2003) is chosen as the local background value for calculating the *I*_geo_ values, which are presented in Table 5. The constant equal 1.5 allows to analyze natural fluctuations in the content of a given substance in the environment and to detect very small anthropogenic influences.

**Table 5:** Geoaccumulation index (*I*_geo_) of heavy metals in urban soils in Toruń

		Cu	Pb	Zn	Ni	Cd	Cr	Hg
Gr. A	Mean	0.1	0.8	−0.9	−2.3	−0.9	−3.9	0.3
	Range	(−3.2)–2.4	(−2.4)–3.4	(−3.3)–0.6	(−4.5)–(−1.1)	(−4.8)–2.2	(−5.1)–(−2.8)	(−4.2)–2.6
Gr. B	Mean	−0.6	−0.3	−0.4	−0.8	−0.3	−1.2	.−0.4
	Range	(−3.1)–(−0.7)	(−2.6)–0.5	(−2.8)–(−0.3)	(−4.1)–(−1.6)	(−3.8)–1.7	(−5.2)–(−3.2)	(−3.0)–0.1
Gr. C	Mean	−0.2	0.8	−0.1	−2.2	−1.0	−3.5	0.6
	Range	(−2.7)–1.5	(−2.2)–2.8	(−2.2)–1.8	(−4.3)–(−1.2)	(−3.2)–0.6	(−5.2)–(−2.2)	(−3.0)–2.9

Gr. A—completely sealed soil, Gr. B—partially sealed soil, Gr. C—non-sealed soil

The average value of *I*_geo_ decreases for soils from group A in the following order: Pb > Hg > Cu > Cd = Zn > Ni > Cr, for soils from group B: Pb = Cd > Hg = Zn > Cu > Ni > Cr, and for soils from group C as follows: Pb > Hg > Zn > Cu > Cd > Ni > Cr. Despite negative values of *I*_geo_ for Cr, Ni, Zn, and Cd in all types of studied soils, they should not be simply classified as “uncontaminated” because such values may be connected with low background concentration of Vistula valley, but not with lack of technogenic accumulation of pollutants. For Pb and Hg *I*_geo_ values in reference soil and completely sealed soil belonged to the category of “uncontaminated to moderately contaminated,” while the average value of *I*_geo_ for partially sealed soil was indicated as “uncontaminated.” This means that the results obtained for each of the elements are considerably different. In the case of Cu, *I*_geo_ in the group of sealed soils had a value higher than zero (“uncontaminated to moderately contaminated”).

Generally, low average values of *I*_geo_ in all examined groups of soil in Toruń have indicated a small level of soil contamination with heavy metals. Simultaneously, it is worth noting that on the basis of the maximum value of *I*_geo_ for Cu, Cd, Pb, Hg, and Zn, sealed soil is classified as “moderately to heavily contaminated.” Moreover, the contamination of Pb and Zn are associated (Fig. 4). It probably means that they have the same source, i.e., cars, but accumulation occurred at different chronological periods, which could be associated with the motor fuel quality (unleaded petrol). Comparison of average *I*_geo_ value among the three groups of soil indicates a following order: partially sealed soil (group B) < non-sealed soil (group C) < completely sealed soil (group A).

### Environmental risk assessment of studied soils

A great number of environmental risk assessment is known in the literature (Hilton et al. 1985; Tomlinson et al. 1980; Verca and Dolence 2005); therefore, the character of the analyzed area was taken into account, and two factors, EF and PLI, were chosen for the evaluation of the soil sealing.

The EF factor evaluated heavy metal pollution according to the content of heavy metals, but it hardly distinguished their source, chemical activity, or biological availability. The main advantage of EF application is the possibility to compare the results with the data presented by other researchers.

Calculation of EF was based on the standardization of a measured element against a reference element. A reference element is often the one characterized by low occurrence variability, such as Al, Fe, Ti, Si, Sr, and K (Duzgoren-Aydin 2007; Sezgin et al. 2003). EF values less than 5.0 are not considered significant, because such small enrichments may arise from differences in the composition of local soil material and reference soil used in EF calculations (Sezgin et al. 2003). However, there is no accepted pollution ranking system or categorization of degree of pollution on the enrichment ratio and/or factor methodology.

The calculated values of EF are presented in Table 6. In the completely sealed soil, average EF values decreased in the following order: Pb > Hg > Cu > Cd > Ni > Zn > Cr, while in the partially sealed soil, as follows: Pb > Cd > Hg > Zn > Cu > Ni > Cr. The average value of the EF for non-sealed soil showed the following diminishing order: Pb > Hg > Zn > Cu > Cd > Ni > Cr, similar to the order described for *I*_geo_. The analysis of EF values have indicated that the content of chromium in all examined groups of soil states “deficiency to minimal enrichment,” whereas the average values of EF for Zn, Ni and Cd have been in the range from 2 to 5, what is called the level of “moderate enrichment.” In the completely sealed soil, the average value of the EF for Hg, Cu, and Pb has been greater than 5, which clearly shows that these elements are derived from anthropogenic sources. In partially sealed soil, only Pb has shown anthropogenic origin, and in the reference soil, average EF index above 5 has been observed for Pb and Cu. It seems, therefore, that EF can also be an effective indicator used to distinguish the natural origin of anthropogenic sources of heavy metals.

**Table 6:** Enrichment factor (EF) and pollution load index (PLI) of heavy metals in urban soils in Toruń

		Cu	Pb	Zn	Ni	Cd	Cr	Hg	PLI
		EF							
Gr. A	Mean	5.1	7.0	2.8	4.0	4.1	0.4	5.4	0.8
	Range	1.0–16.3	1.4–16.4	1.3–6.4	0.7–10.2	0.5–42.7	0.2–0.6	0.4–16.1	0.1–2.3
Gr. B	Mean	2.5	5.5	3.4	1.2	3.8	0.5	3.5	0.4
	Range	1.1–4.4	1.0–20.1	1.1–6.4	0.8–1.6	0.7–25.7	0.4–0.7	0.8–8.6	0.1–1.1
Gr. C	Mean	4.1	7.6	4.4	1.2	2.7	0.5	6.1	0.9
	Range	1.0–9.4	1.1–22.1	1.3–13.8	0.7–1.7	1.0–10.0	0.3–0.6	0.9–22.0	0.2–2.8

Gr. A—completely sealed soil, Gr. B—partially sealed soil, Gr. C—non-sealed soil

The PLI index was evaluated to assess the mutual contamination effects of the seven metals measured in this study.

Mean of PLI (Table 6) was the highest in the non-sealed soils and arranged in the decreasing order as follows: group C > group A > group B, which showed that the non-sealed soils occurred the most contaminated ones. Furthermore, the largest concentrations of pollutants such as polycyclic aromatic hydrocarbons (PAHs) and the greatest biological activity were detected in non-sealed soils in Toruń (Mendyk and Charzyński 2016; Piotrowska-Długosz and Charzyński 2015). The sealing of the soil accompanied limitation of the migration of heavy metals in the soil profile due to the reduction of the supply of the surface levels, limitation of water movement, increase in the specific density of the soil, and the reduction of the degree of aeration. Hence, we can conclude that higher concentrations of Hg, Cu, and Pb have been derived from anthropogenic sources before sealing the soil. It was observed that the content of heavy metals in all soil groups has often been significantly different. This could depend mainly on the type of substrate (e.g., debris, garbage, waste) and the origin of the pollutants such as sewage, sludge, and road traffic, which are characteristics for urban soils (Luo et al. 2012b). Similar conclusions were presented by Bezuglova et al. (2016) who studied sealed and non-sealed soil in Rostov-on-Don. Total contamination factor *Z*_c_ (Revich et al. 1985) was used to show that the sealing of urban soils reduces the vertical migration of heavy metals in the soil profile. The determination of the level of risk of soil contamination with heavy metals depends on their content in the parent rock and on the technogenic deposition in the surface layers. In soils of Rostov-on-Don developed on loess, the content of heavy metals in parent rock was higher than the geochemical background (Bezuglova et al. 2016).

Data were evaluated using classical statistical methods. They did not show a normal distribution according to the Shapiro-Wilk test; thus, they were analyzed using non-parametric Kruskal-Wallis test, but any statistically significant differences were detected. Moreover, PCA of two factors, CA analysis using the Euclidean distance (Lee et al. 2006; Zheng et al. 2008), and Spearman’s correlation coefficients were applied.

**Fig. 3:**
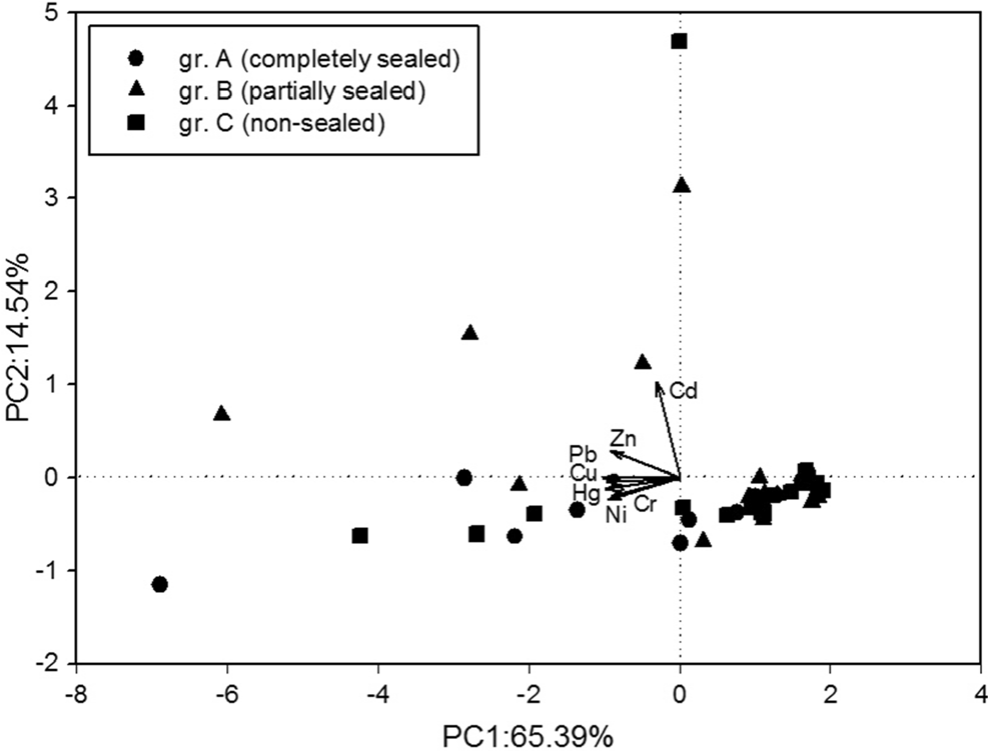
Principal component analysis (PCA) of two factors

**Fig. 4:**
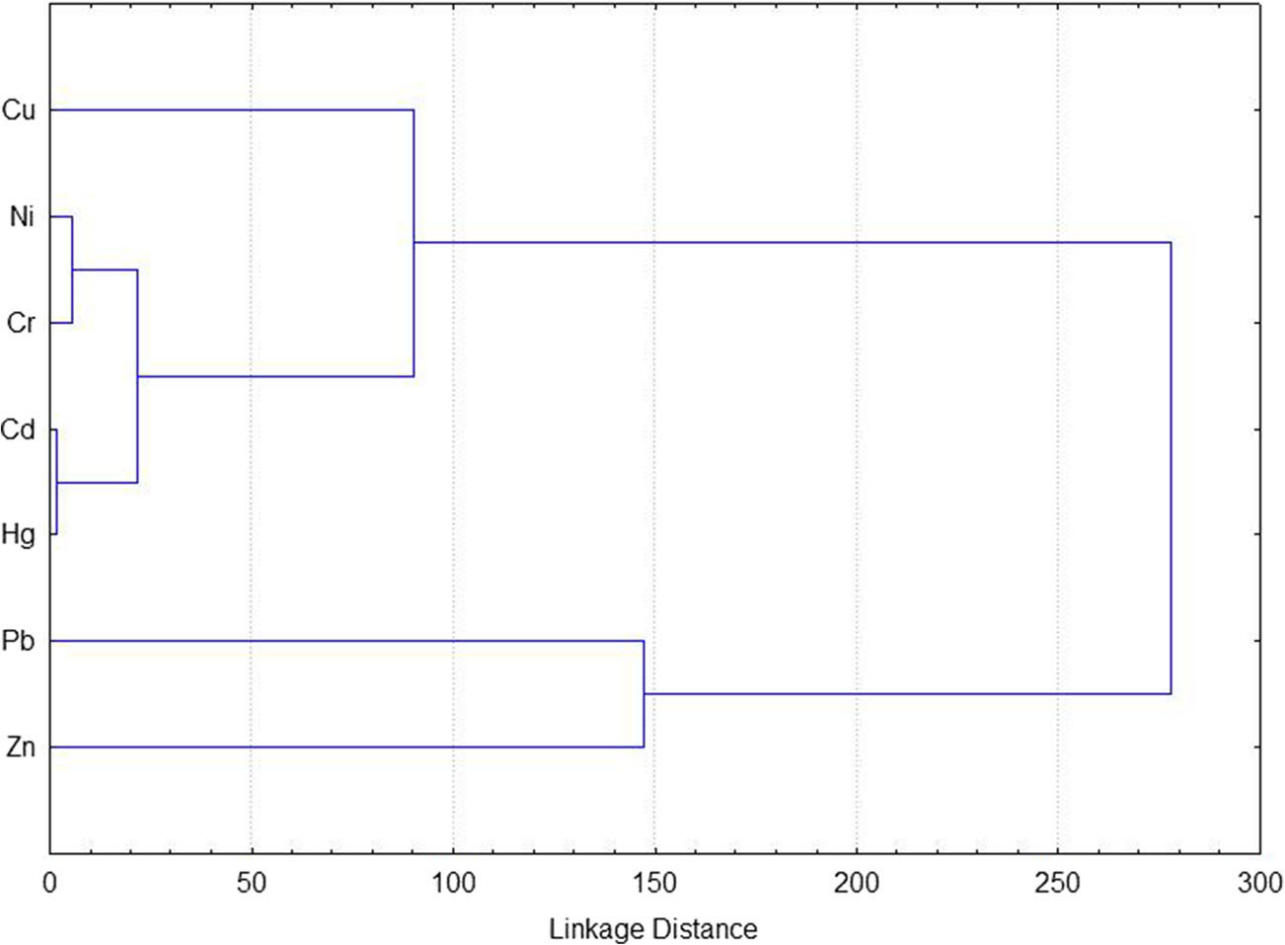
Hierarchical dendrogram for seven heavy metals obtained by cluster analysis (CA)

In PCA analysis, all heavy metals were represented by the first two principal components, which accounted for 79.9% of the total variance. Nevertheless, factors 1 and 2, which explained 65.4 and 14.5% of total variance, respectively, did not differentiate types of soil on the basis of the content of heavy metals (Fig. 3). The result of CA analysis is illustrated in Fig. 4, on which two distinct clusters can be identified. Cluster I contained Cd, Cr, Cu Ni, and Hg, while the long distance between Cu and the other four heavy metals suggested that this cluster could be further divided into two sub-clusters. Cluster II contained Pb and Zn. Concentrations of Cu, Pb, and Hg were significantly higher than the background values of Poland, and concentrations of Cr, Cd, Ni, and Zn were comparable to the background values. Therefore, previously described EF and *I*_geo_ indexes show that the distribution of Cu, Pb, and Hg in urban soils of Toruń was affected mostly by anthropogenic sources, while Cr, Cd, Ni, and Zn were mainly from natural sources. Moreover, significant Spearman’s correlations between heavy metal content and physical properties of the urban soil in Toruń (Table 7) were determined mainly in non-sealed soil and then for completely sealed soil. In partially sealed soil, the amount of correlations was the least. However, partial or complete sealing did not differentiate the soil in terms of heavy metal content, which confirmed the idea that the soil is heterogeneous under technopressure and remains in contrast to the soil formed under natural conditions. Similarly, the results analyzed in the population of non-sealed soil were the evidence of their high heterogeneity. Soil sealing interrupts the exchange between the soil system and other ecological compartments, including the biosphere, hydrosphere, and atmosphere, which affects processes in the water cycle, biogeochemical cycles, and energy transfers (Siebielec et al. 2015). However, in case of the impact of sealing of urban soils on the content of heavy metals, we proved only slight effect.

**Table 7:** The values of Spearman’s correlation coefficients between heavy metal concentrations and physical properties of urban soils in Toruń (significance level 0.05, *p* < 0.05)

	Cu	Pb	Zn	Ni	Cd	Cr	Hg	Sand	Silt	Clay	P	C_org_	N
	(ppm)						(ppb)	(%)			(mg kg^−1^)	(%)	
Gr. A													
Cu	1												
Pb	*0.93*	1											
Zn	*0.86*	*0.86*	1										
Ni	*0.76*	*0.71*	*0.85*	1									
Cd	*0.70*	*0.74*	*0.86*	*0.72*	1								
Cr	*0.73*	*0.74*	*0.89*	*0.91*	*0.72*	1							
Hg	*0.82*	*0.90*	*0.79*	*0.56*	*0.71*	*0.62*	1						
Sand (%)	−0*.*43	−*0.58*	−*0.53*	−*0.54*	−0*.*48	−0*.*45	−0*.*50	1					
Silt (%)	0*.*41	*0.54*	0*.*51	0*.*51	0*.*44	0*.*42	0*.*49	−*0.98*	1				
Clay (%)	*0.59*	*0.70*	*0.69*	*0.70*	*0.52*	*0.70*	*0.70*	−*0.73*	*0.74*	1			
P (mg kg^−1^)	*0.77*	*0.82*	*0.53*	0*.*44	0*.*30	0*.*39	*0.75*	−0*.*41	0*.*38	*0.55*	1		
C_org_ (%)	*0.72*	*0.77*	*0.75*	*0.73*	*0.67*	*0.59*	*0.71*	−*0.67*	*0.62*	*0.71*	*0.64*	1	
N (%)	0*.*48	*0.57*	0*.*50	*0.62*	0*.*43	0*.*46	*0.61*	−0*.*51	0*.*46	*0.71*	*0.61*	*0.85*	1
Gr. B													
Cu	1												
Pb	0*.*55	1											
Zn	*0.66*	0*.*37	1										
Ni	0*.*53	−0*.*21	0*.*27	1									
Cd	*0.85*	0*.*51	*0.81*	0*.*48	1								
Cr	0*.*48	−0*.*14	0*.*12	*0.95*	0*.*39	1							
Hg	0*.*54	*0.74*	0*.*29	−0*.*03	0*.*34	0*.*07	1						
Sand (%)	−*0.67*	−0*.*09	−0*.*32	−*0.83*	−0*.*50	−*0.78*	−1*.*00	1					
Silt (%)	*0.67*	0*.*09	0*.*32	*0.83*	0*.*50	*0.78*	−*0.78*	*0.78*	1				
Clay (%)	0*.*54	−0*.*04	0*.*03	*0.66*	0*.*29	*0.68*	−0*.*34	0*.*34	0*.*05	1			
P (mg kg^−1^)	*0.66*	*0.66*	0*.*54	0*.*14	*0.66*	0*.*17	−0*.*19	0*.*19	0*.*06	*0.55*	1		
C_org_ (%)	0*.*20	*0.65*	0*.*03	−0*.*09	0*.*13	0*.*15	−0*.*19	0*.*19	0*.*12	0*.*30	0*.*56	1	
N (%)	0*.*36	*0.80*	0*.*01	−0*.*05	0*.*32	0*.*13	−1*.*00	*0.78*	0*.*05	0*.*44	0*.*61	*0.86*	1
Gr. C													
Cu	1												
Pb	*0.79*	1											
Zn	*0.84*	*0.80*	1										
Ni	*0.78*	*0.55*	*0.61*	1									
Cd	*0.81*	*0.76*	*0.78*	*0.75*	1								
Cr	*0.78*	*0.54*	*0.61*	*0.96*	*0.66*	1							
Hg	*0.76*	*0.86*	*0.77*	*0.61*	*0.68*	*0.61*	1						
Sand (%)	−*0.65*	−0*.*47	−0*.*50	−*0.88*	−*0.69*	−*0.83*	−*0.63*	1					
Silt (%)	*0.66*	0*.*49	*0.50*	*0.87*	*0.68*	*0.79*	*0.66*	−*0.98*	1				
Clay (%)	*0.65*	0*.*40	*0.54*	*0.87*	*0.51*	*0.85*	*0.57*	−*0.88*	*0.87*	1			
P (mg kg^−1^)	*0.78*	*0.78*	*0.87*	0*.*50	*0.58*	*0.57*	*0.76*	−0*.*42	0*.*42	*0.55*	1		
C_org_ (%)	*0.66*	*0.76*	0*.*41	0*.*45	*0.58*	0*.*41	*0.56*	−0*.*43	0*.*43	0*.*30	*0.55*	1	
N (%)	*0.66*	*0.69*	0*.*39	*0.54*	*0.55*	0*.*47	0*.*51	−*0.55*	*0.56*	0*.*44	0*.*51	*0.96*	1

Significant correlations are printed in italics. Gr. A—completely sealed soil, Gr. B—partially sealed soil, Gr. C—non-sealed soil

The sources of Cd, Cu, Cr, Hg, Ni, Pb, and Zn in urban soils of Toruń are extremely heterogenous, and thus, situation is rather complicated. Anthropogenic sources such as vehicle exhaust, household waste, and construction activities have made the heavy metal concentrations higher than their background values. Besides, as Toruń is a city with a long history, and the historical buildings such as palaces and temples are all well-preserved, the historical use of heavy metals in pigments, wood preservation, and brassware would also play an important role for their accumulation in urban soils around.

## Conclusion

To assess the status of environmental quality in the completely and partially sealed and non-sealed soils, three indicators, *I*_geo_, EF, and PLI, and multifactorial statistical analysis, were used. The results indicate that the artificial sealing in urban areas slightly affects the content of heavy metals in soils. However, based on PLI, it was found that the sealing has influence on soil properties and non-sealed soil is the most exposed to the accumulation of pollutants. Determination of EF index has also become an effective indicator used to distinguish the natural origin from anthropogenic sources of heavy metals. In our studies, it was found that in completely sealed soils, heavy metals (Hg, Cu, Pb) were of anthropogenic origin. Similar pattern was detected for *I*_geo_; thus, for completely sealed soils, it decreased in the following order: Pb > Cu > Hg > Cd > Zn > Ni > Cr, for the partially sealed soil: Pb > Cd > Hg > Zn > Cu > Ni > Cr, while for the reference soil: Pb > Hg > Zn > Cu > Cd > Ni > Cr.
